# Comparing acute effects of extra virgin coconut oil and extra virgin olive oil consumption on appetite and food intake in normal-weight and obese male subjects

**DOI:** 10.1371/journal.pone.0274663

**Published:** 2022-09-16

**Authors:** Ziya Erokay Metin, Pelin Bilgic, Mercan Merve Tengilimoğlu Metin, Muzaffer Akkoca

**Affiliations:** 1 Department of Nutrition and Dietetics, Gulhane Health Sciences Faculty, University of Health Sciences, Ankara, Turkey; 2 Department of Nutrition and Dietetics, Faculty of Health Sciences, Hacettepe University, Sıhhiye, Ankara, Turkey; 3 Department of General Surgery, Dışkapı Yıldırım Beyazıt Training and Research Hospital, University of Health Sciences, Altındağ, Ankara, Turkey; Public Library of Science, UNITED KINGDOM

## Abstract

**Objectives:**

The aim of this study is to compare acute effects of consuming extra virgin coconut oil (EVCO) as a source of medium chain fatty acids and extra virgin olive oil (EVOO) as a source of long chain fatty acids in normal weight and obese subjects.

**Design:**

Randomised, crossover design.

**Participants:**

Metabolically healthy twenty male subjects (10 normal weight; 10 obese) aged 19–40 years.

**Intervention:**

Subjects consumed breakfast meals containing skimmed milk, fat-free white cheese, bread and EVCO (25 g) or EVOO (25 g).

**Outcome measures:**

Visual analog scale evaluations, resting metabolic rate measurements and selected blood parameters analysis (glucose, triglyceride, insulin and plasma peptide YY) were performed before and after the test breakfast meals. In addition, energy intakes were evaluated by *ad libitum* lunch meal at 180 min.

**Results:**

Visual analogue scale values of hunger and desire to eat decreased significantly after EVCO consumption than EVOO consumption in normal weight subjects at 180 min. There was an increase trend in plasma PYY at 30 and 180 min after EVCO breakfast compared to EVOO breakfast. *Ad libitum* energy intakes after EVCO and EVOO consumption in normal weight subjects were 924 ± 302; 845 ± 158 kcal (p = 0.272), respectively whereas in obese subjects were 859 ± 238; 994 ± 265 kcal (p = 0.069) respectively.

**Conclusion:**

The results of this study shows that consumption of EVCO compared to EVOO may have suppressive effect on hunger and desire to eat, may affect postprandial PYY levels differently and have no effect on postprandial energy expenditure.

**Trial registration:**

Clinical Trials NCT04738929.

## Introduction

Obesity is one of the leading health problems worldwide and is associated with cardiovascular diseases, diabetes, musculoskeletal disorders, and certain types of cancer [1]. Excessive consumption of high-energy foods and decreased physical activity patterns are responsible for the global epidemic of obesity [2]. The etiology of obesity is complex and includes genetic, physiological, environmental, psychological, social, and economic factors [3]. However, inappropriate food intake, containing high amounts of fat and carbohydrates, is still the most powerful inducer of obesity [4]. An energy imbalance between intake and expenditure occurs with increasing energy-dense food consumption, resulting in body weight gain [5]. Energy balance is homeostatic, and energy homeostasis maintains the body weight and fat constant by maintaining the balance between energy intake and expenditure in normal-weight individuals [6, 7]. Information on the nutritional status and energy stores is transmitted to the brain through the energy homeostasis process for satiety perception [8]. Therefore, dietary practices targeting satiation/satiety and appetite control might be beneficial in preventing obesity [9]. Epidemiological evidence suggests a direct link between dietary fat intake and obesity [10, 11]. Although high energy consumption from fats leads to weight gain, the types of fat affect the rates differently [12, 13]. Additionally, the different effects of the saturation degree and chain length of fatty acids on fat oxidation have been shown in human studies [14, 15].

Fat is an important part of the diet, and dietary fats commonly include fatty acids with a chain length of 14 carbons or more [16]. Triglycerides (TGs) with chain lengths of 6–12 carbon atoms are medium-chain triglycerides (MCTs), such as capronic acid (C6:0), caprylic acid (C8:0), capric acid (C10:0), and lauric acid (C12:0) [17]. Unlike long-chain triglycerides (LCTs), which are transported by the lymphatic system, MCTs are transported via the portal venous system [18]. MCTs have been proposed as agents for the prevention of obesity, and long-term MCT consumption has been shown to enhance energy expenditure (EE) and fat oxidation in obese women when compared to LCT consumption [19]. In addition, a meta-analysis of randomized controlled trials reported reductions in body weight by replacing dietary LCTs with MCTs [20]. Furthermore, acute studies have found reductions in *ad libitum* energy intake after MCT consumption [17].

Extra virgin coconut oil (EVCO) is a natural source of MCTs, and studies investigating the effects of acute consumption of EVCO on appetite, hormonal response, subsequent energy intake, and EE are scarce [21, 22]. To the best of our knowledge, the acute effects of EVCO on postprandial appetite ratings, biochemical and hormonal parameters, EE, and subsequent *ad libitum* food intake in both metabolically healthy obese and normal-weight subjects have not been previously investigated. Therefore, this study primarily aimed to compare the acute effects of consuming 25 g EVCO containing rapidly oxidized MCTs and extra virgin olive oil (EVOO) as a source of LCTs on postprandial energy expenditure, biochemical parameters (glucose, insulin, TGs, peptide YY [PYY]), satiety, and total energy intake in subsequent meals in normal-weight and obese men following the consumption of EVCO and EVOO.

## Materials and methods

### Study design

This study was planned as a randomized, single-blind, controlled crossover study. Individuals participated in two test sessions on non-consecutive days after the screening session. All of the subjects have taken both EVCO and EVOO treatment. There was a minimum of 1 day and a maximum of 1 week between the test days [22]. We kept the washout period short because long washout period might cause changes in the anthropometric and metabolic values of the subjects. Breakfast meals with EVCO or EVOO were randomly consumed on the test days. A meal containing EVOO was used as a control breakfast. Visual analog scale (VAS) assessments for subjective appetite feelings, energy expenditure measurements, and certain blood parameters (serum glucose, TG, insulin, and plasma PYY hormone) were performed before and after the test breakfast meals. In addition, subsequent energy intake was evaluated at *ad libitum* lunch. This study was registered at ClinicalTrials.gov retrospectively since the importance of registration could not be grasped (Trial number: NCT04738929). The authors confirm that there are no other ongoing or related trials for this intervention.

### Methods

#### Subjects

**Fig 1:**
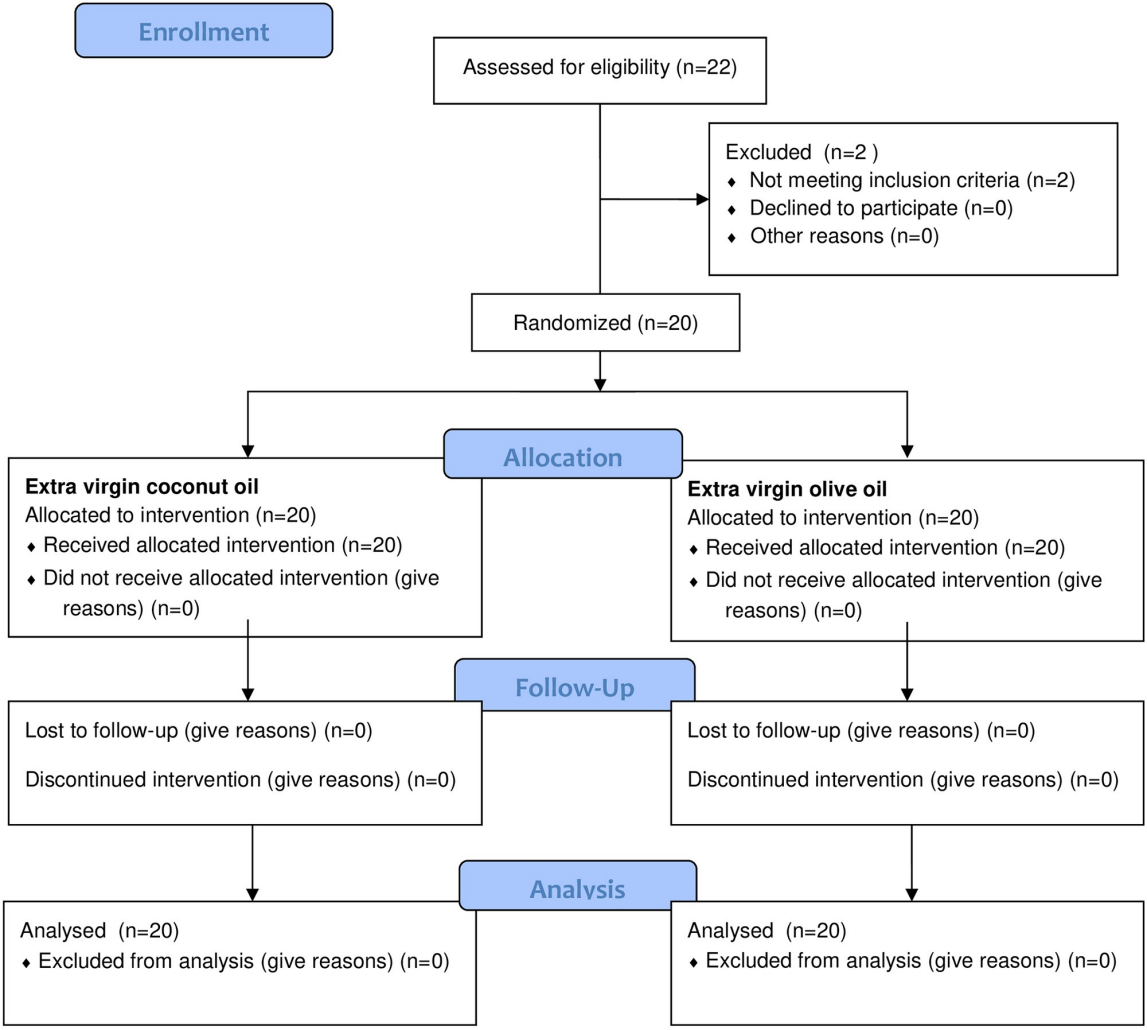
Flow diagram showing process for participants enrollment and randomization.

Twenty metabolically healthy normal-weight (body mass index [BMI] = 18.5–24.9 kg/m^2^) (n = 10) and obese men (BMI = 30–34.99 kg/m^2^) (n = 10) aged 19–40 years were recruited to participate through personal communication and poster advertisements (Fig 1). Volunteers agreed to participate in our study applied to the health center. Volunteers who met the inclusion and exclusion criteria of the study were included in the study according to the order in which they came to the health center with the start of the study. Only male subjects were included to eliminate potential variations due to hormonal changes during the menstrual cycle. Detailed information about the study was provided to the men who agreed to participate, and a health questionnaire was administered for a history of food allergies/intolerances, diseases, medication, and smoking. The Three-Factor Eating Questionnaire and International Physical Activity Questionnaire were completed by the subjects to determine eating behaviors and physical activity patterns. The exclusion criteria were smoking, alcohol consumption, weight gain/loss recently (>%5, in 3 months), any genetic or metabolic diseases, any food allergy/intolerance, any medication, and restrained eating habits. Subjects included in the study were asked to maintain their usual eating habits and physical activity patterns.

#### Procedure

The subjects visited the clinic thrice; the first was for the screening and the other two for the actual testing. During the screening session, body weight and height were measured. The day before each test day, the subjects were asked not to exercise and to record their food intake at dinner, and to consume a similar meal on the night before the second test day.

Simple randomization was conducted for the order in which participants take the treatments [21]. Some participants have taken EVCO treatment first, and the others have taken EVOO treatment first. Subjects assigned to each treatment purely randomly for every assignment. On the test days, the subjects arrived at the laboratory at 8 a.m. Upon arrival, the baseline satiety level was evaluated using VAS, and blood samples were taken for biochemical and hormonal parameters, and the resting metabolic rate (RMR) was measured after the subjects rested briefly. After the fasting assessments, the subjects were fed with a breakfast meal containing EVCO or EVOO within 20 min. The subjects were screened for the subsequent 3 h until lunch was served to measure *ad libitum* energy intake. During the test sessions, the subjects were allowed to visit the toilet. All subjects received two test breakfast meals in random order.

#### Test breakfast meals

The subjects consumed standard breakfast meals containing 25–30% of their daily energy requirements as test meals. The standard breakfast meal comprised fat-free cow milk (300 mL), white bread (75 g), fat-free feta cheese (30 g), and EVCO (25 g) or EVOO (25 g). The subjects soaked their bread with EVCO or EVOO and consumed them without a break after consuming milk and cheese. The energy and nutrient values of the breakfast meal were calculated using the Nutrition Information System Program (BEBIS 11.3; Turkish version, Pasifik Company) (Table 1). The fatty acids composition of Extra Virgin Olive Oil (EVOO) and Extra Virgin Coconut Oil (EVCO) was well documented in the literature and studies have indicate that the MCT content of EVCO is clearly different from the MCT content of EVOO [23–26]. The findings obtained from our study have been discussed considering the fatty acid profile contents of EVCO and EVOO reported in previous studies.

**Table 1:** Energy and macro nutrients composition of test breakfast meals.

Foods	Energy (kcal)	CHO (g)	Protein (g)	Fat (g)
Fat-free milk (300 ml)	108	15	10.5	0.3
Fat-free cheese (30 g)	26	0.6	5.2	0.7
White bread (75 g)	192	39.7	6.1	0.7
EVCO / EVOO (25 g)	221 / 223	-	-	24.9
Total	546 / 549	55.3	21.8	26.6

The daily energy requirement was determined according to Schofield equation and the physical activity by the International Physical Activity Questionnaire. There are different methodologies in previous studies examining the acute effects on appetite and metabolism. However, studies on coconut oil have focused more on a certain dose. In this study the dose of the fats was chosen not to affect the health negatively and would have influence on appetite ratings. It has been reported in a recent study that acute consumption of 25 ml coconut oil, which is a reasonable amount, does not show negative collateral effect of high-fat meals [21]. On the other hand, 26 g of coconut oil has shown to reduce food intake throughout the day [22].

#### Analyses

*Anthropometric measurements*. Body weight was measured with the subjects wearing light clothing and without shoes, and height was measured using a stadiometer during the screening visit. BMI was calculated from the weight and height of the subjects.

*EE*. RMR and postprandial energy expenditure were measured before and after the test breakfast meals at 0, 60, 120, and 180 min using a Cosmed Fitmate Pro indirect calorimeter (Cosmed, Italy). The Fitmate Pro has a turbine flow meter for measuring ventilation and an oxygen sensor for analyzing the oxygen in the expired gases. A face mask was placed over the subject’s face and attached to the turbine flow meter to sample the expired air. Between the measurement periods subjects were told to lie quietly and small amount of movement and reading was permitted. At 10 minutes before the start of each measurement subjects were instructed to rest motionless. The Fitmate Pro also self-calibrates before each measurement (5 min) during the 15-min RMR assessment. Data of the first 5 minutes of the measurement was excluded from the analysis [27].

*Subjective appetite feelings*. A 10 cm continuous line VAS was used to determine the subjective ratings for hunger, fullness, and the desire to eat [28]. Subjects were instructed to place a vertical line on the intervals of the VAS to indicate their ratings, and the VAS score was evaluated at 0, 30, 60, 120, and 180 min. The most negative and positive ratings were anchored at the end of the scale. The VAS comprised three questions: 1) How hungry do you feel? 2) How full do you feel? 3) How strong is your desire to eat? The subjects filled in separate papers at each time point. All papers were stapled and collected in the end. The subjects answered the VAS alone.

*Blood sampling and biochemical analysis*. Blood samples were obtained before and after the test breakfast meals at 0, 30, 60, 120, and 180 min after VAS measurements, and the same row was applied in all subjects and at all measurement points. Serum and plasma samples were collected with a cannula inserted into an antecubital vein, which was kept patent by flushing with saline. Blood samples were collected in ethylenediamine tetra-acetic acid-coated chilled tubes for plasma PYY and serum tubes for serum glucose, insulin, and TGs. Serum and plasma samples were separated from whole blood by centrifugation (4000 rpm, 4°C, 10 min) and immediately frozen at -80°C until analysis. Commercially available enzyme-linked immunosorbent assays (ELISA) kits were used to measure serum glucose (Human Glucose ELISA kit—Sunred, 201-12-8477), insulin (Human Insulin ELISA kit—Elabscience, E-EL-H2665), TGs (Human TG ELISA kit—Sunred, 201-12-2086), and plasma PYY (Human Peptide YY ELISA kit—Elabscience, E-EL-H1237). All analyses were performed in duplicate.

*Ad libitum food intake*. An *ad libitum* sandwich lunch was chosen to measure the energy intake at the end of the 3-h test session, as suggested by Blundell et al. [29]. Three sandwiches were served in small pieces (two pieces from one bread) to subjects at once, and they were asked to continue eating another piece after each piece was consumed until they felt comfortably full. The subjects were warned before lunch to avoid consuming certain parts. The leftover food was weighed after the breakfast meal to determine the total food intake. The same sandwiches were served to the subjects on both test days. The sandwiches comprised cheddar cheese, tomatoes, and white bread. The energy and nutrient values of the sandwiches served at lunch and breakfast were calculated using the Nutrition Information System Program (BEBIS 11.3; Turkish version, Pasifik Company) (Table 2).

**Table 2:** Energy and macro nutrient composition of per sandwich served at lunch.

Foods	Energy (kcal)	CHO (g)	Protein (g)	Fat (g)
Cheese (60 g)	255	0	11.5	23.5
White bread (100 g)	256	52.9	8.1	0.9
Tomato (50 g)	9	1.3	0.5	0.1
Total	520	54.2	20.1	24.5

#### Statistical analyses

Data analyses were performed using IBM SPSS Statistics 20, and the significance value was set at p<0.05. Values are presented as the mean ± standard deviation. A power analysis was performed using the Gpower 3.1.9.2 package program, with VAS as the primary outcome. This power calculation showed that a sample size of 20 subjects would have a power of 71% with 0.92 effect size and type I error of 0.05. Data normality was assessed using the Shapiro-Wilk test, and data approximating the normal distribution were considered to be normally distributed. The effects of the oil type and groups on postprandial parameters were assessed using repeated-measures analysis of variance in the mixed model, and time and treatments (oil type) were included in the model. The areas under the curves (AUCs) were calculated for EE, VAS, serum glucose, insulin, TGs, and plasma PYY values using the trapezoidal rule for AUC by summarizing the mean scores of the pairs of adjacent time points and ‘repeated analysis of variance’ was used for comparing the AUC.

#### Ethical statement

The study protocol was approved by the Ethics Committee of Hacettepe University (Protocol number: KA-17163) and conducted in accordance with the Declaration of Helsinki. All subjects provided written informed consent after receiving verbal and written information.

## Results

Twenty metabolically healthy male individuals were enrolled in this study. The two groups of the study comprised 10 normal-weight and 10 obese subjects. The characteristics of the subjects are presented in Table 3. All subjects completed each of the two test days.

**Table 3:** Baseline measurements of subjects.

Parameters	Normal weight (n = 10)	Obese (n = 10)
Age (year)	33.5 ± 3.9	35.5 ± 4.1
Body weight (kg)	76.0 ± 5.8	95.0 ± 5.5
Height (cm)	177 ± 7.1	174 ± 4.7
BMI (kg/m^2^)	24.1 ± 0.7	31.2 ± 1.5
Body fat percentage (%)	18.8 ± 2.7	25.4 ± 2.6
RMR (kcal/day)	1622 ± 151	1863 ± 201
Glucose (mg/dl)	85.9 ± 6.4	90.6 ± 8.8
Insulin (mmol/L)	8.6 ± 5.4	12.3 ± 7.8
PYY (mg/dl)	224.4 ± 103.1	213.7 ± 126.8
TG (mg/dl)	127.6 ± 59.7	146.1 ± 46.6

Data was presented as mean ± standard deviation. Metabolic variables are averaged from baseline values of each participant at EVCO and EVOO test days

### EE

The effects of the test breakfasts on EE measures are shown in Table 4. There was no treatment effect for EE in both normal-weight and obese subjects (p>0.05). In addition, the AUCs for EE were not different between the test oils and groups (S1 Fig).

**Table 4:** Absolute changes of EE following the consumption of EVCO and EVOO consumption in NW and O subjects.

Energy expenditure	Time (min)			
	0	60	120	180
**Normal weight (n = 10)**				
EVCO (kcal)	1585.4±140.8	1916.1±275.7	1918±189.1	1765.3±197.6
EVOO (kcal)	1660.3±160.3	1849.7±224.4	1923±208.9	1854.5±245.1
**Obese (n = 10)**				
EVCO (kcal)	1870.5±198.1	2183.5±222.5	2139±213.9	1941.5±285
EVOO (kcal)	1856.5±216.2	2094.5±284.9	2028.2±265.4	1905.1±276.1

Data was presented as mean ± standard deviation.

### Biochemical measurements

The mean values of the participant baseline biochemical measurements are presented in Table 3. The AUC values of glucose were significantly higher in obese subjects than in normal-weight subjects after the consumption of both test breakfasts (p<0.05) (S2 Fig). The EVOO breakfast increased postprandial glucose values more than the EVCO breakfast in both normal-weight and obese subjects; however, there was no significant effect of the treatment (p>0.05) (S2 Fig). The AUC values for insulin were significantly different between the two groups after the consumption of EVOO (p<0.05). There was no effect of treatment on postprandial insulin levels in normal-weight and obese subjects (p>0.05). There was no significant effect of the oil type on postprandial PYY levels in both normal-weight and obese subjects (p>0.05). Absolute changes in all biochemical measurements following the consumption of the test breakfasts in normal-weight and obese subjects are shown in Table 5.

**Table 5:** Absolute changes of biochemical parameters of normal weight and obese subjects following the consumption of coconut oil and olive oil consumption in normal weight and obese subjects.

Biochemical parameters		Normal weight					Obese				
		Time (min)					Time (min)				
		0	30	60	120	180	0	30	60	120	180
Glucose (mg/dL)	EVCO	88.1±6.5	102.3±14.2	85.4±1	91.2±8.9	86.5±9.1	91±9.5	118.1±13.5	116.3±25.7	99.3±14.4	92.4±11.8
	EVOO	83.8±5.9	101.1±9.7	89.1±13.5	88.6±14.5	86±14	90.2±8.5	115.3±13.4	119.5±21.7	105.9±19.1	92.9±13.7
Insulin (mU/L)	EVCO	10.32±6.1	65.4±32.2	43.7±19.7	42.6±26.6	16±15.3	10.8±8.2	72.8±42.9	82.6±51.9	50.3±39.4	25.3±28.6
	EVOO	6.93±4.2	44.1±33.1	36.7±29.8	36.9±28.4	15.5±20.5	13.8±69.4	69.4±34.7	94.2±38.1	57.8±34.6	28.1±30.4
PYY (pg/dL)	EVCO	203±69.9	244.9±104.3	255.5±152.2	231.3±90.9	250.7±123.6	206.7±140.1	205.4±105.5	189.7±92.1	189.6±95.6	205.2±101.5
	EVOO	245.8±128.5	244±92.8	261.9±96.9	253.7±114	240.8±103.5	220.7±119.2	216.9±106.8	213.8±113.3	217.5±113.3	215.1±108
TG (mg/dL)	EVCO	126.8±61.8	141.8±66.7	119.1±62.6	117.9±58	152.4±120.5	146.1±51.1	149.9±51.1	147.6±54.9	155.3±57.2	163.6±57.2
	EVOO	128.4±60.9	189.6±232.2	179.6±227.9	198.9±230.8	220.4±278.4	146.1±47.5	151.6±54.1	154.7±50.6	139.7±45.9	

Data was presented as mean ± standard deviation.

### Subjective appetite feelings

The EVCO breakfast caused significantly lower hunger and desire to eat responses when compared with the EVOO breakfast at 180 min in normal-weight subjects (p<0.05); however, this difference was not observed in obese subjects (p>0.05) (S3 Fig). There was no significant effect of the oil type on the AUC for subjective appetite feeling scores (p>0.05).

### *Ad libitum* food intake

There was no significant effect of the oil type on the *ad libitum* energy intake of normal-weight subjects (EVCO, 924±302 kcal vs. EVOO, 845±158 kcal; p = 0.272). Obese subjects had a lower *ad libitum* energy intake after the consumption of the EVCO breakfast compared with the EVOO breakfast (EVCO, 859±238 kcal vs. EVOO, 994±265 kcal); however, this difference was not statistically significant (p = 0.069).

## Discussion

Studies evaluating the effects of EVCO consumption on hunger/satiety hormones are scarce, and new randomized controlled human studies are needed to provide nutritional recommendations. This is the first study to investigate the effects of EVCO consumption on the gut hormone PYY and food intake in both obese and normal-weight individuals.

We showed that EVCO has more suppressive properties than EVOO on hunger and the desire to eat at 180 min only in normal-weight men. In addition, there was an increasing trend in plasma PYY after the EVCO breakfast compared with the EVOO breakfast; however, these results do not match with the *ad libitum* energy intake. On the other hand, significant differences in postprandial glucose and insulin levels between normal-weight and obese men were observed.

### EE

The effects of different fatty acids on postprandial energy expenditure have been previously studied [30–32]. Flint et al. showed no difference in the postprandial energy expenditure between monounsaturated, polyunsaturated, and trans fatty acids in overweight men [30]. In contrast, Casas-Agustench et al. reported that meals containing monounsaturated and polyunsaturated fatty acids increased postprandial energy expenditure compared with a meal containing saturated fat [33]. A study comparing the acute effects of MCT oil and corn oil as an LCT showed higher postprandial oxygen consumption after the MCT oil challenge [34]. In addition, higher postprandial energy expenditure was observed after MCT oil consumption according to the LCT consumption [35]. These studies were conducted with an MCT oil; however, studies assessing the effects of EVCO as a source of MCT are scarce in humans. Valente et al. reported that coconut oil did not affect postprandial resting energy expenditure differently than EVOO in a study on female individuals with high body fat rates [21]. Another study comparing coconut oil and corn oil showed no significant acute effects on postprandial energy expenditure in obese adolescents [36]. Similar to previous studies performed with coconut oil, resting energy expenditure in the acute period was not affected by the type of oil.

### Biochemical measurements

#### Glucose and insulin

There is a strong link between postprandial hyperglycemia after a glucose challenge and the risk of cardiovascular death and all-cause mortality [37]. Postprandial increase in glucose and TG levels cause excess free radical formation, and when this happens multiple times daily, it can result in atherosclerotic risk factors [38]. Fat addition to bread as a breakfast challenge has been shown to reduce postprandial glycemic response without a significant difference in the fat type [39]. In addition, Clegg et al. reported significantly different glycemic responses when healthy volunteers consumed different types of fats with carbohydrate-containing meals, and the postprandial glycemic responses were lower than the control challenge [40]. In contrast, a 21-day MCT diet intervention compared with an LCT diet resulted in higher plasma glucose concentrations after MCT, and the insulin concentrations did not differ between the diets [41]. In contrast, Khaw et al. reported no significant changes in blood glucose levels within a 4-week intervention with coconut oil, olive oil, and butter [42]. A recent study comparing the acute effects of coconut oil and olive oil intake showed no significant effect on the total postprandial glucose and insulin levels [21]. Similarly, we found no significant differences in glucose and insulin levels between the test oils. In addition, the EVCO breakfast had a lower AUC_glucose_ than the EVOO breakfast challenge in obese and normal-weight subjects. Additionally, the EVCO breakfast resulted in a lower subsequent energy intake than the EVOO breakfast in obese subjects (p = 0.069) but not in normal-weight subjects (p = 0.272). Therefore, our results suggest a better glucose response and satiating effect for EVCO than EVOO in obese subjects. However, this result suggests that a better blood glucose response does not always mean a lower subsequent energy intake. The negative correlation between blood glucose and *ad libitum* energy intake has been supported by other studies [43, 44].

Decreased insulin sensitivity is a well-known complication of obesity [45]. In addition, decreased insulin sensitivity in overweight subjects could cause differences in appetite regulation between normal-weight and overweight subjects. As a satiety factor, insulin may also promote further weight gain in insulin-resistant subjects [46]. Flint et al. suggested that postprandial insulin levels are associated with acute appetite regulation in normal-weight subjects; however, this interaction disappears as the body weight increases, and they proposed that insulin resistance might explain the blunted effect on appetite [47]. In our study, obese subjects had a significantly higher AUC_glucose_ than normal-weight subjects for the two breakfast challenges. On the other hand, AUC_insulin_ was significantly different between normal-weight and obese subjects when they consumed the EVOO breakfast; however, this difference was not observed for the EVCO breakfast. Although there were differences between glucose and insulin responses in normal-weight and obese subjects, these results did not match the *ad libitum* energy intake after the EVCO breakfast. Normal-weight subjects had a higher *ad libitum* energy intake than obese subjects after the EVCO breakfast, whereas obese subjects had a higher energy intake after the EVOO breakfast. These confusing outcomes suggest that the oil type influences the postprandial state differently between normal-weight and obese subjects.

#### PYY

Gut peptides that are known to influence satiety are released after the entry of fat into the small intestine [48]. PYY is one of these hormones, which has been shown to be stimulated by both MCT and LCT infusion intraduodenally [49]. On the other hand, a greater release of PYY with the consumption of MCTs than LCTs has been reported in overweight men [50]. In our study, we found no effect of the oil type on postprandial PYY release in normal-weight and obese subjects.

#### TGs

Postprandial TG levels have been reported to be predictors of future myocardial infarction risk [51] and are known to increase after standard meal consumption in normal-weight individuals [52]. Furthermore, Kasai et al. showed significantly lower postprandial TG levels after an MCT meal than an LCT meal in subjects with a BMI of ≥23 kg/m^2^ but not in subjects with a BMI of <23 kg/m^2^ [53]. Our study demonstrated a greater increase in postprandial TG levels in normal-weight subjects after EVOO consumption; however, this difference was not statistically significant.

#### Subjective appetite feelings and *ad libitum* energy intake

The decrease in hunger and desire to eat scores at 180 min after consumption of the EVCO breakfast meal relative to a breakfast comprising EVOO was found to be significantly higher in normal-weight subjects. However, these differences were not observed for AUCs of hunger and the desire to eat evaluated using VAS. On the other hand, EVCO consumption caused higher *ad libitum* energy intake than EVOO consumption in normal-weight subjects; however, this difference was not statistically significant. Antithetically, *the ad libitum* energy intake of obese subjects after the consumption of the EVOO breakfast meal was higher than that of the EVCO breakfast meal. Studies with a larger sample size might create significantly different results for *ad libitum* energy intake between EVCO and EVOO in obese subjects. Van Wymelbeke et al. showed decreased subsequent food intake after breakfasts containing MCTs than LCTs [54]. Likewise, Rolls et al. reported a 14% decrease in the energy intake of non-dieters with MCT consumption vs. LCT consumption [55]. Another study showed a decreasing energy intake at dinner after MCTs than LCTs with added lunch [56]. In contrast, Poppit et al. reported no difference in the energy intake between short-chain TGs, MCTs, and LCTs [57]. This difference might be due to studies reporting significantly different results using 20 g or more MCT oil; however, Poppit et al. used 10 g MCT oil. Contrary to these findings, a recent study comparing the effects of MCT oil (25 g) and coconut oil (26 g) on energy intake showed that MCT oil decreased energy intake in subsequent meals more than coconut oil. In the same study, coconut oil consumption led to lower energy intake throughout the day compared with LCTs [22]. Another study reported no difference in the appetite and energy intake between coconut oil and sunflower oil [58]. Although we observed favorable effects of EVCO compared with EVOO on appetite in normal-weight subjects, this positive effect did not match the *ad libitum* energy intake.

Our study had several strengths. First, our study used a crossover design. Second, we included both metabolically healthy normal-weight and obese subjects. In addition, we measured the postprandial energy expenditure to compare the metabolic effects of EVCO and EVOO. On the other hand, it is one of the few studies in which EVCO was used.

Our study has certain limitations. In this study, a small sample size and exclusion of women subjects might have caused a lack of statistically significant differences between the test meals and groups On the other hand, we screened subjects for 3 h; however, the relatively long (>3 h) time-lapse between breakfast and *ad libitum* lunch might show better eating behavior and one day/meal data may not show overall outcomes. Washout period between treatments could be same among subjects but there was a minimum of 1 day and a maximum of 1 week between the test days. Based on previous studies, 25 g of coconut oil was used in this study, which may not be suitable for a single meal [21, 22]. In addition, overall nutrient and fatty acid profiles of test breakfasts were not analyzed.

## Conclusion

In conclusion, the results of our study suggest that acute consumption of EVCO in mixed breakfast has suppressive effects on hunger in normal-weight men.

Recently, new promising strategies to improve weight loss have attracted the attention of researchers. Our findings showed a suppressive effect of EVCO on subsequent energy intake in obese subjects and may potentially contribute to weight loss. Additionally, this report provides evidence that 25 g of EVCO consumption has different acute effects on metabolism when compared with 25 g of EVOO. Therefore, these results encourage further research on appetite and food intake modulation in weight management strategies, and long-term studies with larger samples are also crucial to determine the effects of EVCO consumption.

## Supporting information

S1 Fig AUCs for EE measures following the consumption of EVCO and EVOO consumption in NW and O subjects. (TIF)

S2 Fig AUCs for glucose, insulin, PYY, TG measurements following the consumption EVCO and EVOO in NW and O subjects. (TIF)

S3 Fig Absolute changes from baseline and AUCs for hunger (A), fullness (B), desire to eat (C) responses following the consumption of EVCO and EVOO in NW and O subjects. (TIF)

S1 File Data set. (XLSX)

S1 Checklist (DOC)

S1 Graphical abstract (TIF)

S1 Protocol Study protocol (English). (DOCX)

S2 Protocol Study protocol (Turkish). (DOCX)
