# Supplementary figures and images for: Comparing acute effects of extra virgin coconut oil and extra virgin olive oil consumption on appetite and food intake in normal-weight and obese male subjects
Source: PLoS One. 2022 Sep 16;17(9):e0274663. doi: 10.1371/journal.pone.0274663 (PMC9480981; doi:10.1371/journal.pone.0274663)

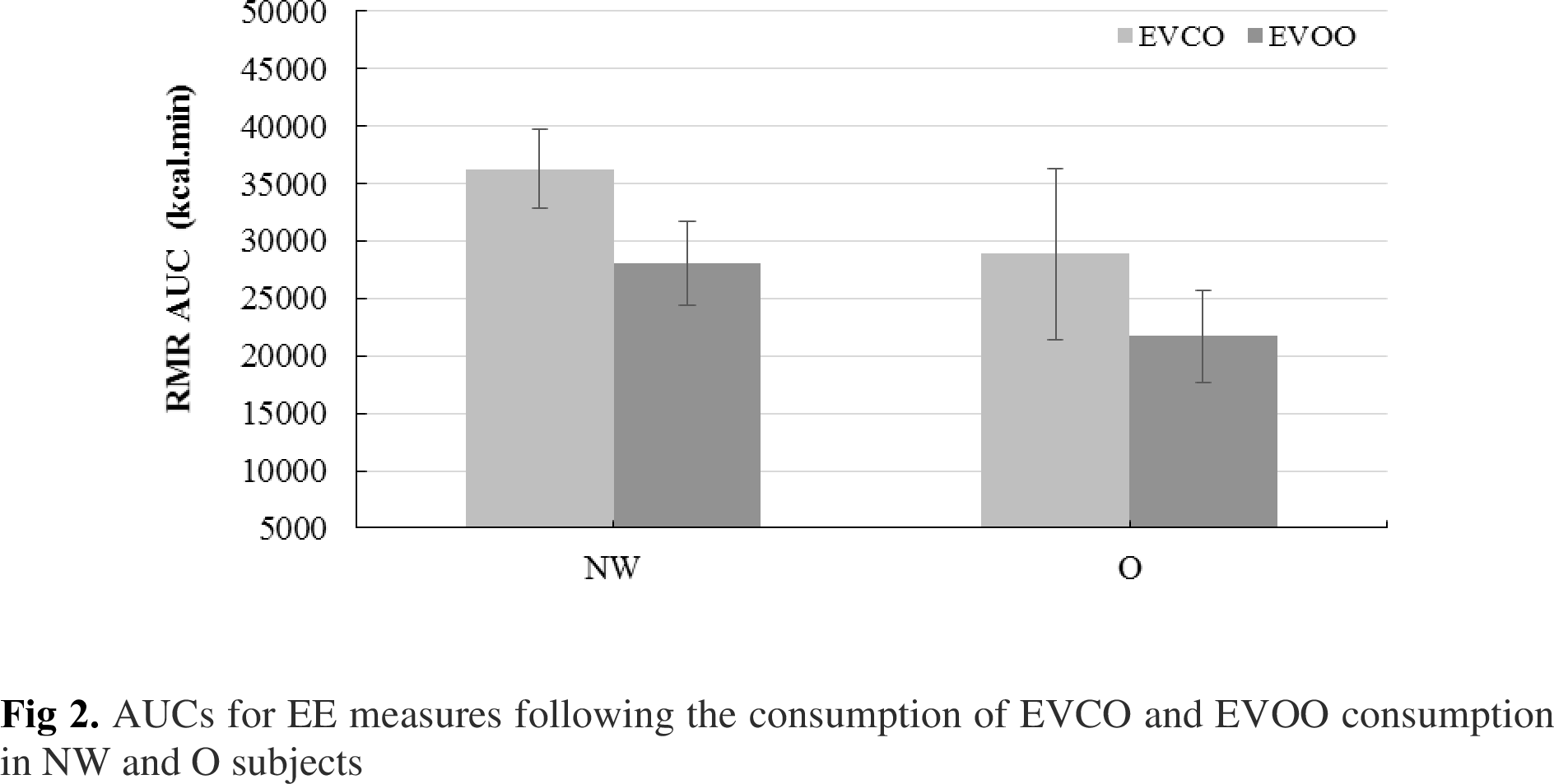

Supplement: S1 Fig — (TIF) [file pone.0274663.s001.tif]

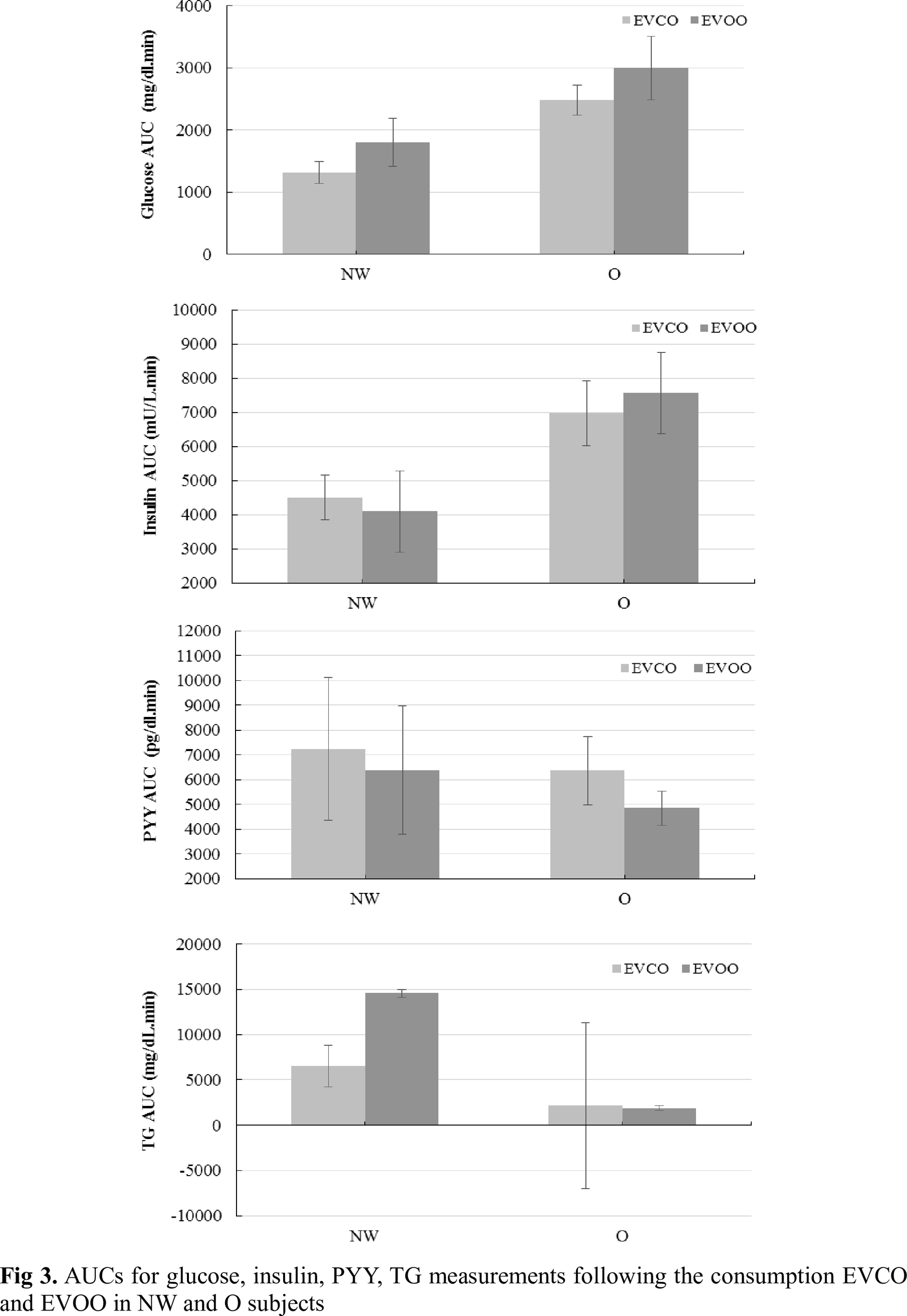

Supplement: S2 Fig — (TIF) [file pone.0274663.s002.tif]

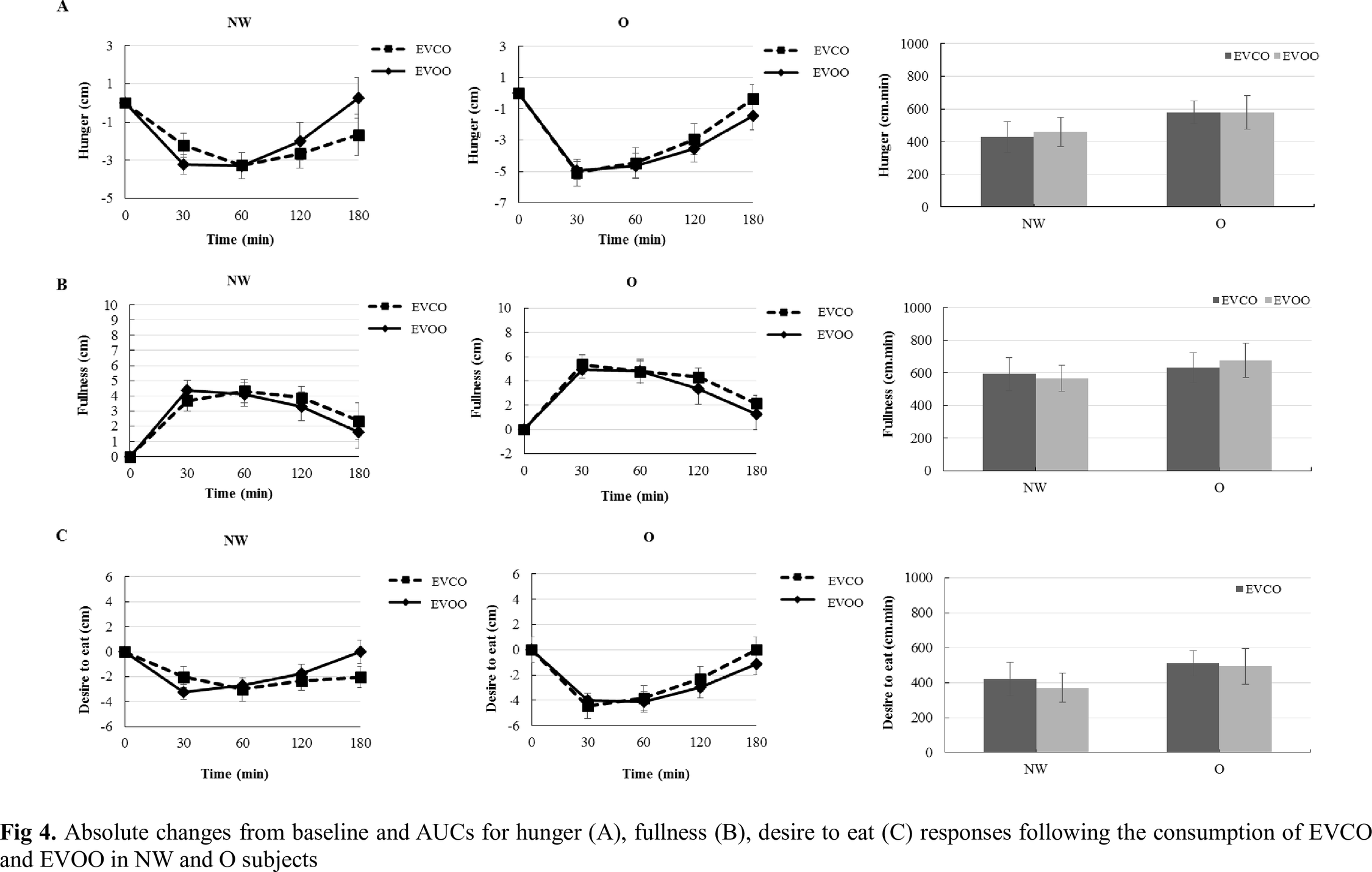

Supplement: S3 Fig — (TIF) [file pone.0274663.s003.tif]

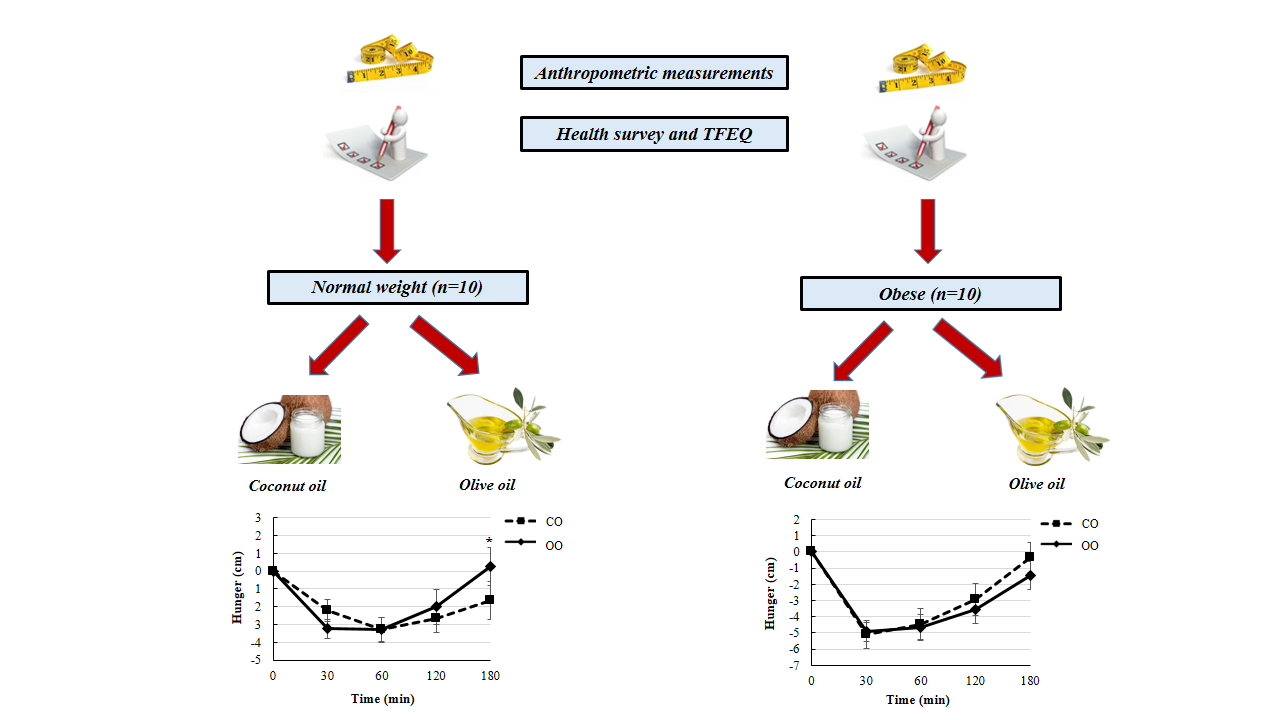

Supplement: S1 Graphical abstract — (TIF) [file pone.0274663.s006.tif]
