# Supplementary material for: Comparing acute effects of extra virgin coconut oil and extra virgin olive oil consumption on appetite and food intake in normal-weight and obese male subjects
Source: PLoS One. 2022 Sep 16;17(9):e0274663. doi: 10.1371/journal.pone.0274663 (PMC9480981; doi:10.1371/journal.pone.0274663)
Supplement: S1 Protocol — (DOCX) [file pone.0274663.s007.docx]

**STUDY PROTOCOL**

This study was planned as a randomized single-blind controlled crossover. Individuals will attend two test sessions on non-consecutive days after the screening visit. There will be a minimum of one day and a maximum of one week between test days. Breakfast meal containing coconut oil or olive oil will be consumed by individuals on test days. A meal containing olive oil will be taken as the reference group. VAS evaluations, energy expenditure measurements, analysis of some blood parameters (glucose, triglyceride, insulin and plasma peptide YY hormone) will be performed before and after the test breakfast meal. In addition, energy intakes after the ad-libitum lunch will be evaluated.

Individuals who agree to participate in the study will be evaluated according to the study inclusion and exclusion criteria after signing the informed consent form. A 'three-factor nutrition questionnaire' will be applied to determine the eating behaviors of individuals and an 'international physical activity questionnaire' will be applied to determine physical activity levels. A health questionnaire questioning food allergy and intolerance, presence of genetic or metabolic disease, drug use and smoking will be administered. 10 normal weight and 10 obese male individuals will be included in the study. Women will not be included in the study due to possible effects that may occur due to hormonal changes during the menstrual cycle.

**Inclusion criteria**

- Male 19-40 years old

- Normal weight (BMI 18.5 – 24.9 kg/m2) and obesity (30-34.99 kg/m2)

**Exclusion criteria**

- Cigarette consumption

- Alcohol consumption

- Recent weight change (> 5%, three months)

- Family history or chronic disease in individuals (hypercholesterolemia, cardiovascular diseases, glucose intolerance or Diabetes, liver diseases, renal diseases)

- Genetic and metabolic disease

- Food allergy or food intolerance to any food

- Use of drugs known to affect energy expenditure or gastrointestinal functions

- Individuals showing restricted eating habits in the three-factor nutrition questionnaire evaluation

Individuals included in the study will be asked to maintain their current lifestyle and physical activity and avoid consuming foods containing coconut oil.

Individuals will come to the Health Center on three different days to perform a screening visit one day and a test session two days. At the screening visit, body weight and height will be measured after 8 hours of fasting.

There will be a minimum of 1 day and a maximum of 1 week between test days. In the days between test days, individuals will be asked to continue their usual eating habits. Subjects will be asked to fast for 12 hours on the day of the test, not to have taken medication in the last 24 hours, and not to have done any physical activity in the last 48 hours.

Individuals will come to the clinic hungry at 08:00 in the morning and from 20:00 in the previous evening. Subjects will be asked to record the previous dinner and consume the same dinner before the next test day. At 08:30, initial hunger and satiety visual analog scale (VAS) assessments, energy expenditure and body weight measurements will be made. Individuals will be given 200 ml of drinking water and an intravenous cannula will be inserted and blood samples will be taken. After assessments made while fasting, individuals will be asked to sit upright for 10 minutes before the test meal is given. At 09:00, individuals will be asked to consume a breakfast meal containing coconut oil or olive oil within 15-20 minutes. VAS evaluations and blood sampling will be repeated at 09:30, 10:00, 11:00, and 12:00. Energy consumption measurements will be made at 10:00, 11:00, 12:00. Ad libitum lunch will be served at 12:00. During the testing session, individuals will be allowed to sit, read, walk quietly (also for a short time outside the dining room), listen to the radio without food images or narration, or watch TV/video. They will be made to go to the toilet when they need it. Individuals will not be allowed to sleep during the test session. All individuals will receive both test meals in random order.

Individuals will consume a standard breakfast containing 25-30% of their energy requirements as a test meal. In case the standard breakfast meal does not meet the energy needs of individuals, the amounts of nutrients will be increased in equal proportions (except for test oils). The standard breakfast meal will include skim cow's milk (300 ml), white bread (75 g), nonfat feta/curd cheese (30 g), extra virgin coconut oil (25 g) or virgin olive oil (25 g). Virgin coconut oil and virgin olive oil will be protected from light and heat until consumption. Skimmed cow's milk will be served below 10oC.

Body weight measurements of individuals will be made during the screening visit and test days while the individual is hungry, with shorts, no shoes and no socks. Height measurement will be measured with a stadiometer at the screening visit. Body mass index (BMI) will be calculated from the weight and height values of the individuals.

Individuals' hunger and satiety will be assessed with a valid VAS questionnaire. In the VAS questionnaire used in this study, individuals' hunger, satiety, desire to eat and how much they think they can eat will be questioned with the most positive and the most negative scoring. Situations for each of the questions in the questionnaire will be measured on a fixed 100 mm horizontal line at both ends. Individuals will be taught to place a vertical line on the 100 mm line according to how they feel at the moment. The measurement is quantified by measuring the distance from the left end of the line to the mark. Microsoft Office Excel 2013 package program will be used in the calculation of the area under the curve of the VAS score graphs of the individuals. VAS questionnaires will be administered before the test meal and at 30., 60., 120., 180. minutes after the start of the test meal consumption.

Energy expenditure will be measured by indirect calorimetry before the test meal and at the 30th, 60th, 120th, 180th minutes after the start of the test meal consumption.

Blood samples will be taken by inserting an intraket into the individuals by venous blood collection method. The anterior surface of the elbow and the inner part of the arm, where there are large veins close to the skin surface, are the preferred sites for venous blood collection (antecubital fossa). Before the consumption of the test meal and at the 30th, 60th, 120th and 180th minutes after the test meal consumption, 12 ml of blood samples, 12 ml each, will be taken into EDTA tubes and serum tubes. After centrifugation (20 min, 4oC) it will be stored at -20oC until analysis. Serum glucose, triglyceride, insulin and plasma peptide YY hormone analyzes in blood samples will be performed by ELISA method.

Individuals will consume lunch ad libitum 3 hours after the start of test meal consumption. In order to measure their nutritional intake, individuals will be asked to consume sandwiches containing cheddar cheese as lunch until they are full. The weight of the sandwiches consumed by the individuals will be measured before and after consumption, and the energy intake of the individuals will be determined by measuring the weight of the remaining amount. Individuals will be served the same sandwiches on both test days and given 20 minutes for an ad libitum lunch. Individuals will also be served 200 ml of drinking water and their consumption will be recorded.

When individuals reach the target number (n=10+10=20) in both groups (coconut oil and olive oil) according to inclusion and exclusion criteria, and data from individuals in the study protocol (blood samples, VAS assessments, energy expenditure measurements) Once collected, the study will be terminated.
