# Supplementary material for: Comparing acute effects of extra virgin coconut oil and extra virgin olive oil consumption on appetite and food intake in normal-weight and obese male subjects
Source: PLoS One. 2022 Sep 16;17(9):e0274663. doi: 10.1371/journal.pone.0274663 (PMC9480981; doi:10.1371/journal.pone.0274663)

**ARAŞTIRMA PROTOKOLÜ**

1. **Araştırmanın Adı:**

Sağlıklı ve Obez Bireylerde Hindistan Cevizi Yağı ve Zeytinyağı Alımının Açlık ve Tokluk Üzerine Akut Etkilerinin Değerlendirilmesi

1. **Araştırmanın Gerekçesi:**

Obezite önemli bir halk sağlığı sorunudur (1). Bireylerin beslenme alışkanlıklarındaki farklılıklar toplumda obezite yaygınlığının artmasına neden olan önemli bir faktördür. Obezite genel olarak enerji içeriği yoğun, şeker ve doymuş yağ asitlerinden zengin besinlerin tüketimi ve sedanter yaşam tarzı ile karakterizedir (2). Yağdan zengin beslenmenin ağırlık artışı ve obezite ile ilişkisi, yapılan çalışmalarda gösterilmiştir. Sadece tüketilen yağın miktarı değil aynı zamanda türünün de adipoz doku depolarını etkileyen önemli bir faktör olduğu bildirilmiştir (3).

Yağlar önemli bir enerji kaynağıdır. Yağda çözünen vitaminlerin emiliminde elzem olması nedeniyle günlük kalori alımının % 30’ u yağlardan sağlanmalıdır. Çoğu yağlı besinlerin enerji içeriği yoğundur ve lezzetlidir, ancak protein ve karbonhidrattan zengin besinlerle karşılaştırıldığında tokluk üzerine etkisi daha zayıf kalmaktadır. Bundan dolayı yağdan zengin beslenme ağırlık artışına ve obeziteye neden olabilir. Yağlardan kaynaklanan ağırlık artışını önlemenin bir yolu besinlerden alınan yağın miktarını azaltmaktır. Bu yöntem besinlerin lezzetini azaltarak tüketimi de azaltmaktadır. Başka bir yaklaşım ise besinlerin yağ içeriğini devam ettirerek yağların türünü değiştirmek olabilir. Örnek olarak, farklı karbon zinciri uzunluğunda veya doygunluk seviyesinde yağların kullanımı emilim mekanizmalarını etkileyebilir. Bu durum besinlerin lezzetini azaltmazken aynı zamanda tokluk durumunu etkileyerek sonraki öğünlerde besin alımlarını etkileyebilir. Farklı zincir uzunluklarına sahip yağların emilimleri ve metabolizmaları da farklıdır. Orta zincirli trigliseritler daha küçük molekül ağırlığına sahip olmalarından dolayı uzun zincirli yağ asitlerine göre daha hızlı hidrolize olurlar. Şilomikronlar tarafından taşınan ve lenfatik sisteme giren uzun zincirli trigliseritlerden farklı olarak orta zincirli trigliseritler portal sisteme girerler, karaciğere daha hızlı ulaşırlar ve keton cisimciklerinin oluşumuna neden olurlar. Hepatik oksidasyon ve keton cisimciklerinin varlığı besin alımlarında azalma ile ilişkilendirilmektedir, bu durum orta zincirli yağ asitlerinin uzun zincirli yağ asitlerine göre besin alımlarını daha fazla azaltacağı hipotezini desteklemektedir (4).

Deney hayvanları üzerinde yapılan çalışmalarda yüksek orta zincirli yağ asidi içeren diyetlerin besin alımını azalttığı gösterilmiştir (5). Ayrıca, Wiley ve Leveili orta zincirli yağ asitlerinin zayıflamada kullanılabileceğini bildirmiştir (2). Orta zincirli yağ asitleri hızlı hepatik oksidasyona girerler ve düşük seviyede depolanırlar ve bu yağ asitlerinin hem obez hem de ideal ağırlıktaki bireylerde enerji harcamasını, yağ oksidasyonunu ve tokluğu artırdığı, enerji alımını azalttığı gösterilmiştir. Ancak bazı yapılan çalışmalarda orta zincirli yağ asitlerinin tüketilmesiyle postprandiyal yağ asit oksidasyonu ve enerji harcamasındaki artışın ad libitum enerji alımlarına önemli etki göstermediği bildirilmiştir (3).

Obezite prevelansının artışı nedeniyle obezite tedavisinde farklı yöntemler ortaya çıkmaktadır. Merkezi sinir sisteminin besin alımı regülasyonunda yağ asit metabolizmasının enerji dengesi üzerindeki etkisi nedeniyle önemli rol oynadığını düşünülmektedir. Merkezi sinir sistemi besinlerin sindirimi ve enerji harcanmasının kontrolünden sorumludur. Besin alımının uyarımı ve inhibasyonu kısa dönem, vücut yağ depolarının kontrolü uzun dönem mekanizmalarla merkezi sinir sistemi tarafından kontrol edilmektedir. Makro besin öğelerinin dağılımı tokluğu etkilemektedir. Ek olarak diyette yer alan lipitlerin özelliklerinin doyurucu potansiyeli etkilediği düşünülmektedir. Flint ve ark. lipitlerin kompozisyonlarına göre farklı metabolik yolaklarının olduğunu ve iştahı farklı yönde etkilediklerini bildirmiştir (2). Ek olarak, ex vivo çalışmalar orta zincirli yağ asit oksidasyonunun uzun zincirli yağ asit oksidasyonuna göre 10 kat daha hızlı olduğunu göstermiştir. Orta zincirli trigliseritlerin tüketimi uzun zincirli trigliseritlerin tüketimine göre daha fazla termik etki sağlamaktadır, insan ve hayvanlarda tokluğu artırmaktadır. Orta zincirli trigliserit tüketiminin bu etkileri düşünüldüğünde uzun zincirli yağ asitlerinin yerine zayıflama programlarında yardımcı olarak kullanılabileceği hipotezlenebilir (6).

Yapılan çalışmalar orta zincirli trigliseritlerin uzun zincirli trigliseritlere göre ağırlık yönetiminde tercih edilebileceğini göstermektedir. Ancak saflaştırılmış orta zincirli yağ asit yağı ulaşılabilir değildir ve ticari olarak pahalıdır. Hindistan cevizi yağı orta zincirli yağ asitlerini doğal olarak bulunduran en iyi kaynaktır. Bu nedenle hindistan cevizi yağı içeren öğünlerin insanlar üzerine etkilerinin incelenmesi sağlık açısından önemlidir (7). Ek olarak, on iki hafta boyunca uzun zincirli ve orta zincirli yağ asitleri içeren yağın vücut ve adipoz doku ağırlığında önemli seviyelerde azalma sağladığı bildirilmiştir. Orta zincirli trigliseritler direk portal dolaşıma emilirler ve karaciğere ulaşırlar. Karaciğerde β-oksidasyon ile metabolize olurlar. Buna karşın uzun zincirli yağ asitleri bağırsaktan emilir ve şilomikronlar ile dolaşıma katılır. Dolaşımda lipoprotein lipazlar uzun zincirli yağ asitlerini monogliserit ve yağ asitleri gibi daha küçük parçalara ayırırlar. Ayrıca kas dokular tarafından oksidasyona uğrayabilirler veya adipoz dokuda depolanabilirler (2). Hindistan cevizi yağının % 94’ ü doymuş yağdır. Hindistan cevizi yağının hızlı okside olan orta zincirli yağ asit içeriği (doymuş yağın % 32’ si, % 51.6 laurik asit) postprandiyal yağ oksidasyonunu artırarak termogenezi artırabilir. Bu etkiler uzun dönemde kardiyometabolik riskleri azaltabilir ve ağırlık kaybını artırabilir. Orta zincirli trigliseritlerin glikoz kadar hızlı emildiği ve metabolize olduğu kabul edilmektedir (1). Bu bilgiler Hindistan cevizi yağının obez bireylerde tedavi amacıyla kullanılabilecek bir öğe olduğunu desteklemektedir (2). Orta zincirli trigliseritlerin önemli bir kaynağı olan Hindistan cevizi yağı tüketimi ile hormonal düzeyde besin alımlarını inceleyen çalışmaya rastlanmamıştır. Hindistan cevizi yağı tüketimi ile tokluk, besin alımları ve iştah düzenleyici hormonlar arasındaki ilişkilerin incelenmesi orta zincirli yağ asitlerinden zengin bu yağın obezitenin tıbbi beslenme tedavisinde kullanılabilirliğine ışık tutacaktır.

1. **Araştırmanın Amacı:**

Orta zincirli yağ asitlerinin kaynağı olan hindistan cevizi yağı tüketiminin zeytinyağı tüketimine göre akut dönemde enerji harcamasını, metabolik parametreleri ve enerji alımını farklı etkilediğinin gösterilmesi amaçlanmaktadır.

1. **Araştırmanın Tasarımı:**

Bu çalışma, randomize tek kör kontrollü çapraz geçişli olarak planlanmıştır. Bireyler tarama viziti sonrası birbirini takip etmeyen günlerde iki test seansına katılacaktır. Test günleri arasında en az bir gün, en fazla bir hafta olacaktır. Test günlerinde hindistan cevizi yağı veya zeytinyağı içeren kahvaltı öğünü bireylere tükettirilecektir. Zeytinyağı içeren öğün referans grup olarak alınacaktır. Bireylerin test kahvaltı öğünü öncesi ve sonrası VAS değerlendirmeleri, enerji harcaması ölçümleri, bazı kan parametreleri analizleri (glikoz, trigliserit, insülin ve plazma peptid YY hormonu) yapılacaktır. Ayrıca *Ad-libitum* öğle öğünü sonrası enerji alımları değerlendirilecektir.

1. **Araştırmanın Metodu**

**5.1. Çalışma Popülasyonu**

T.C. Cumhurbaşkanlığı Genel Sekreterliği çalışanlarına mail listesi yoluyla ulaşılacaktır ve ilgilenenler çalışmaya davet edilecektir. İlgilenen bireylere çalışma ile ilgili ayrıntılı bilgi verilecektir, gönüllü olur formu okutulacaktır. Çalışmaya katılmayı kabul eden bireyler, bilgilendirilmiş gönüllü olur formunu imzaladıktan sonra çalışma dahil olma ve dışlama kriterlerine göre değerlendirilecektir. Bireylerin yeme davranışlarının belirlenmesi için ‘üç faktörlü beslenme anketi’ ve fiziksel aktivite düzeylerini belirlemek için ‘uluslararası fiziksel aktivite anketi’ uygulanacaktır. Besin alerjisini ve intoleransını, genetik veya metabolik hastalık varlığını, ilaç kullanımını ve sigara tüketimini sorgulayan sağlık anketi uygulanacaktır. Çalışmaya 10 normal ağırlıktaki ve 10 obez erkek birey alınacaktır. Kadınlar menstrual döngü sırasındaki hormonal değişikliklerinden kaynaklı oluşabilecek olası etkilerden dolayı çalışmaya dahil edilmeyecektir.

***Dahil olma kriterleri***

- 19-40 yaş arası erkek
- Normal ağırlık (BKİ 18,5 – 24.9 kg/m^2^) ve obezite (30-34,99 kg/m^2^)

***Dışlama kriterleri***

- Sigara tüketimi
- Alkol tüketimi
- Yakın zamanda ağrılık değişimi (> %5, üç ay)
- Aile öyküsünde veya bireylerde kronik hastalığı (hiperkolesterolemi, kardiyovasküler hastalıklar, glikoz intoleransı veya Diyabet, karaciğer hastalıkları, renal hastalıklar)
- Genetik ve metabolik hastalığı
- Herhangi bir besine karşı besin alerjisi veya besin intoleransı
- Enerji harcaması veya gastorintestinal fonksiyonları etkilediği bilinen ilaç kullanımı
- Üç faktörlü beslenme anketi değerlendirmesinde kısıtlı yeme alışkanlığı gösteren bireyler

Çalışmaya dahil edilen bireylerden mevcut yaşam biçimlerini ve fiziksel aktivitelerini sürdürmeleri ve hindistan cevizi yağı içeren besinleri tüketmekten kaçınmaları istenecektir.

**5.2. Çalışma Protokolü**

Bireyler, Sağlık Merkezine bir gün tarama viziti, iki gün test seansını gerçekleştirmek üzere üç farklı gün gelecektir. Tarama vizitinde 8 saatlik açlık sonrası vücut ağırlığı ve boy uzunluğu ölçülecektir.

Test günleri arasında en az 1 gün, en fazla 1 hafta olacaktır. Test günleri arasındaki günlerde bireylerden olağan beslenme alışkanlıklarına devam etmeleri istenecektir. Bireylerden test günü için 12 saatlik açlık, son 24 saat içinde ilaç almamış olmaları ve son 48 saatte fiziksel aktivite yapmamış olmaları istenecektir.

Bireyler kliniğe sabah saat 08:00’de bir önceki akşam saat 20:00’den itibaren aç olarak gelecektir. Bireylerden bir önceki akşam yemeğini kaydetmeleri ve diğer test gününe gelirken aynı akşam yemeğini tüketmeleri istenecektir. Saat 08:30’da başlangıç açlık ve tokluk görsel analog skala (VAS) değerlendirmeleri, enerji harcaması ve vücut ağırlığı ölçümleri yapılacaktır. Bireylere 200 ml içme suyu verilecektir ve intravenöz kanül takılacak ve kan örnekleri alınacaktır. Aç olarak yapılan değerlendirmelerden sonra, test öğünü verilmeden önce bireylerin 10 dakika dik bir şekilde oturmaları istenecektir. 09:00’da bireylerden hindistan cevizi yağı veya zeytinyağı içeren kahvaltı öğününü 15-20 dakika içerisinde tüketmeleri istenecektir. Saat 09:30, 10:00, 11:00, 12:00’de VAS değerlendirmeleri ve kan örnekleri alımı tekrarlanacaktır. Saat 10:00, 11:00, 12:00’de enerji harcaması ölçümleri yapılacaktır. Saat 12:00’de *ad libitum* öğle yemeği servis edilecektir. Test seansında bireylerin oturmalarına, kitap okumalarına, sessizce yürümelerine (yemek odasının dışında da kısa bir süre için), besin görseli veya anlatımı olmayan radyo dinlemelerine veya TV/video izlemelerine izin verilecektir. İhtiyaç duyduklarında tuvalete gitmeleri sağlanacaktır. Bireylerin test seansı sırasında uyumasına izin verilmeyecektir. Tüm bireyler, rasgele sırayla iki test öğününü de alacaktır.

**5.3. Test kahvaltı öğünü**

Bireyler, test öğünü olarak enerji gereksinimlerinin %25-30’unu içeren standart kahvaltı tüketecektir. Standart kahvaltı öğününün bireylerin enerji ihtiyacını karşılamaması durumunda besinlerin miktarları eşit oranlarda artırılacaktır (test yağları hariç). Standart kahvaltı öğünü, yağsız inek sütü (300 ml), beyaz ekmek (75 g), yağsız beyaz peynir/lor peynir (30 g), natürel sızma hindistan cevizi yağı (25 g) veya natürel sızma zeytinyağı (25 g) içerecektir. Natürel sızma hindistan cevizi yağı ve natürel sızma zeytinyağı tüketime kadar ışık ve ısıdan korunacaktır. Yağsız inek sütü 10^o^C’nin altında servis edilecektir.

**Tablo 1.** Hindistan cevizi yağı/zeytinyağı içeren test öğününün enerji ve besin öğeleri içerikleri

| **Besinler** | **Enerji (kkal)** | **CHO (g)** | **Protein (g)** | **Yağ (g)** |
| --- | --- | --- | --- | --- |
| Yağsız inek sütü (300 ml) | 108,3 | 15 | 10,5 | 0.3 |
| Yağsız beyaz peynir / lor peynir (30 g) | 25,5 | 0,6 | 5,2 | 0,7 |
| Beyaz ekmek (75 g) | 191,8 | 39,7 | 6,1 | 0,7 |
| Hindistan cevizi yağı / zeytinyağı (25 g) | 220,5 - 223 | - | - | 24,9 |
| Toplam | 546,1 - 548,6 | 55,3 | 21,8 | 26,6 |

**5.4. Analizler**

**5.4.1. Antropometrik Ölçümler**

Bireylerin vücut ağırlığı ölçümleri tarama vizitinde ve test günlerinde birey aç iken şort ile ayakkabısız ve çorapsız olarak yapılacaktır. Boy uzunluğu ölçümü, tarama vizitinde stadiometre ile ölçülecektir. Bireylerin ağırlık ve boy değerlerinden beden kütle endeksleri (BKI) hesaplanacaktır.

**5.4.2. Görsel Analog Skalalar (VAS)**

Bireylerin açlık ve doygunluk durumları, geçerli bir VAS anketi ile değerlendirilecektir. Bu çalışmada kullanılan VAS anketinde bireylerin açlık, doygunluk, yeme isteği ve ne kadar yiyebileceğini düşündükleri en olumlu ve en olumsuz puanlama ile sorgulanacaktır. Ankette yer alan her sorunun her birine ilişkin durumlar, her iki ucu sabit 100 mm'lik bir yatay çizgi üzerinde ölçülecektir. Bireylere, o anki zamanda nasıl hissettiklerine göre 100 mm'lik çizgiye dikey bir çizgi yerleştirmeleri öğretilecektir. Ölçümün niceliği, çizginin sol ucundan işarete olan mesafeyi ölçerek yapılır. Bireylerin VAS skorları grafiklerinin eğri altı alan hesaplamalarında Microsoft Office Excel 2013 paket programı kullanılacaktır. VAS anketleri, test öğünü öncesi ve test öğünü tüketiminin başlangıcından sonraki 30., 60., 120., 180. dakikalarda uygulanacaktır.

**5.4.3. Enerji harcaması**

Enerji harcaması, test öğünü öncesi ve test öğünü tüketiminin başlangıcından sonraki 30., 60., 120., 180. dakikalarda indirek kalorimetre ile ölçülecektir.

**5.4.4. Kan örneklerinin toplanması ve analizleri**

Kan örnekleri venöz kan alma yöntemi ile bireylere intraket takılarak alınacaktır. Cilt yüzeyine yakın ve geniş venlerin bulunduğu dirseğin ön yüzü ve kolun iç kısmı venöz kan almada tercih edilecek bölgedir (antekübital fossa). Bireylerden test öğünü tüketimi öncesi ve test öğünü tüketim başlangıcından sonraki 30., 60., 120., 180. dakikalarda 12’ şer ml olmak üzere toplam 60 ml kan örnekleri EDTA’lı tüplere ve serum tüplerine alınacaktır. Santrifüj (20 dakika, 4^o^C) işleminden sonra analize kadar -20^o^C’de depolanacaktır. Kan örneklerinde serum glikoz, trigliserit, insülin ve plazma peptid YY hormon analizleri, ELISA yöntemi ile yapılacaktır.

**5.4.5. Ad-libitum öğle öğünü (Besin alımı)**

Bireyler test öğünü tüketiminin başlangıcından 3 saat sonra *ad libitum* öğle öğünü tüketeceklerdir. Besin alımlarının ölçülmesi için bireylerden öğle öğünü olarak kaşar peynir içeren sandviçlerden doyuncaya kadar tüketmeleri istenecektir. Bireylerin tükettikleri sandviçlerin tüketim öncesi ve tüketim sonrası ağırlıkları ölçülecektir, kalan miktarın ağırlığı tekrar ölçülerek bireylerin enerji alımları belirlenecektir. Her iki test gününde de bireylere aynı sandviçler servis edilecektir ve bireylere *ad libitum* öğle öğünü için 20 dakika verilecektir. Bireylere ayrıca 200 ml içme suyu servis edilecektir ve tüketimler kaydedilecektir.

**Tablo 2.** Öğle öğününde servis edilecek sandviçlerin enerji ve besin öğleri değerleri

| **Besinler** | **Enerji (kkal)** | **CHO (g)** | **Protein (g)** | **Yağ (g)** |
| --- | --- | --- | --- | --- |
| Kaşar peynir (60 g) | 255,1 | 0 | 11,5 | 23,5 |
| Beyaz ekmek (100 g) | 255,7 | 52,9 | 8,1 | 0,9 |
| Domates (50 g) | 8,7 | 1,3 | 0,5 | 0,1 |
| Toplam | 519,5 | 54,2 | 20,1 | 24,5 |

**5.4.6. İstatistiksel Analizler**

Tüm verilerin analizi için SPSS 20.0 istatistik paket yazılımı kullanılacaktır. İki müdehale (hindistan cevizi yağı ve zeytin yağ) grubuna göre her VAS sorusu, kan glukoz, insülin ve peptid YY hormon analizleri için ortalama, aralık, standart sapma ve standart hata içeren tanımlayıcı istatistikler hesaplanacaktır. Kan glukozu, trigliserit, insülin, peptid YY hormon düzeyleri ve VAS soruları için eğri altındaki kalan alan, trapezoid yöntem kullanılarak hesaplanacaktır. Kan glukozu, trigliserit, insülin, peptid YY hormon düzeyleri ve VAS soruları için iki müdehale grubu arasında belirgin farklılıkların olup olmadığını belirlemek için tekrarlı ölçümlerde varyans analizi (ANOVA) kullanılarak yapılacaktır. İstatistiksel değerlendirmeler için p <0.05 olarak belirlenecektir.

**5.5. Araştırma Sonlanım Noktası:**

Bireyler, dahil olma ve dışlama kriterlerine göre her iki grupta da (hindistan cevizi yağı ve zeytin yağ) hedeflenen sayıya (n=10+10=20) ulaştığında ve bireylerden çalışma protokolünde yer alan veriler (kan örnekleri, VAS değerlendirmeleri, enerji harcaması ölçümleri) toplandığında çalışma sonlandırılacaktır.

**Kaynaklar**

1. Valente, F.X., Cândido, F. G., Lopes, L., L. Effects of coconut oil consumption on energy metabolism, cardiometabolic risk markers, and appetitive responses in women with excess body fat. European Journal of Nutrition (2017).
2. Costa, A. C., Rosado, E. L., Soares-Mota, M. Influence of the dietary intake of medium chain triglycerides on body composition, energy expenditure and satiety; a systematic review. Nutricion Hospitalaria (2011) 27; 103-108.
3. Kinsella, R., Maher, T., Clegg, M.E. Coconut oil has less satiating properties than medium chain triglyceride oil. Physiology & Behavior (2017) 179; 422–426
4. Rizzo, G., Masic, U., Harrold, J. A. Coconut oil and sunflower oil ratios in ice cream influence subsequent food selection and intake. Physiology & Behavior (2016) 164; 40 - 46
5. Poppitt, S.D., Strik, C.M., MacGibbon, A.K.H. Fatty acid chain length, postprandial satiety and food intake in lean men. Physiology & Behavior (2010) 101; 161–167.
6. St-Onge, M., Mayrsohn, B., Keeffe, M. Impact of medium and long chain triglycerides consumption on appetite and food intake in overweight men. Eur J Clin Nutr. (2014) 68(10): 1134–1140.
7. Bhavsar, N., St-Onge, M. The diverse nature of saturated fats and the case of medium-chain triglycerides: how one recommendation may not fit all. Curr Opin Clin Nutr Metab Care (2016) 19: 81–87.

**Çalışmanın Şematik Gösterimi**

**
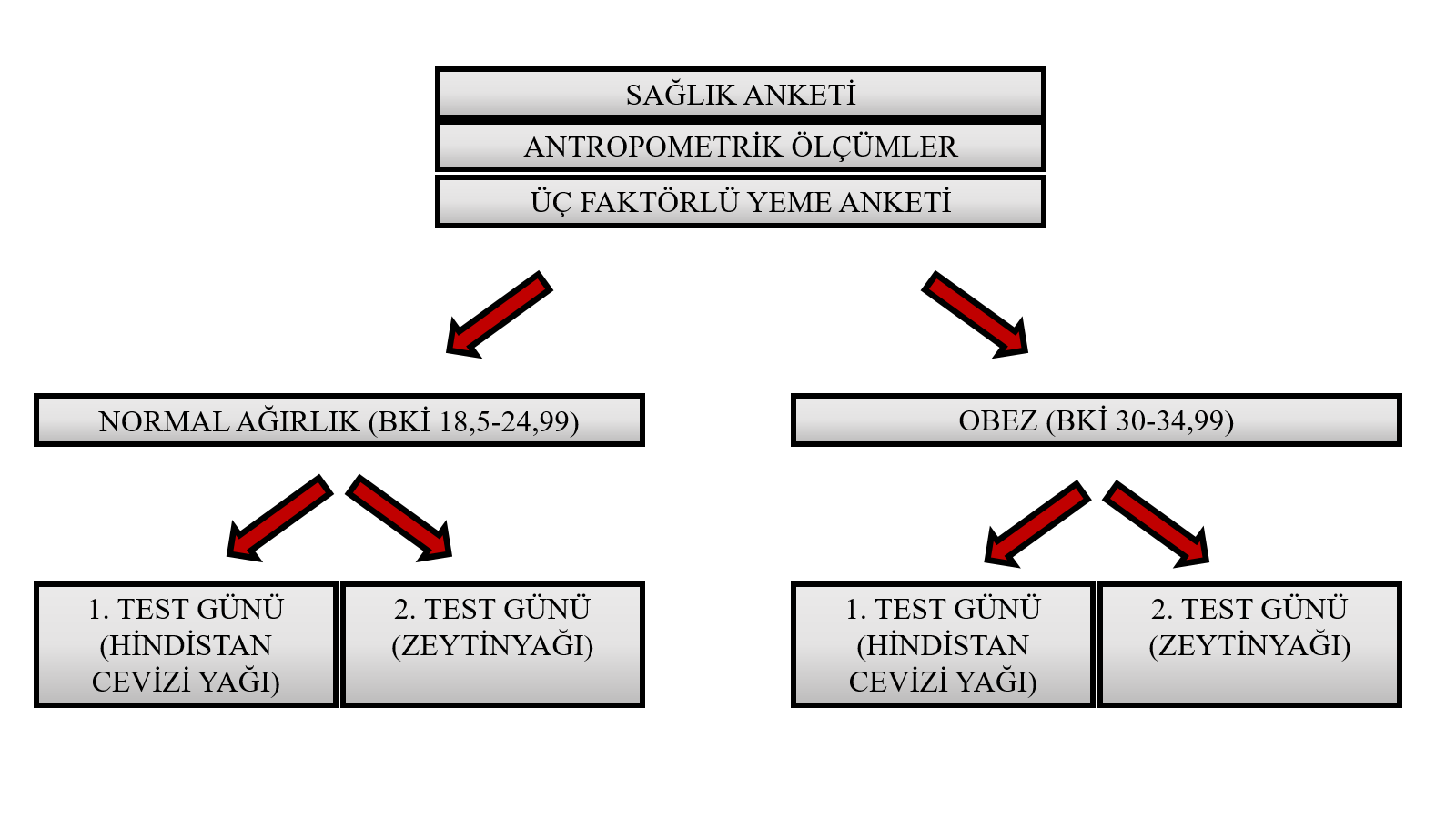
**


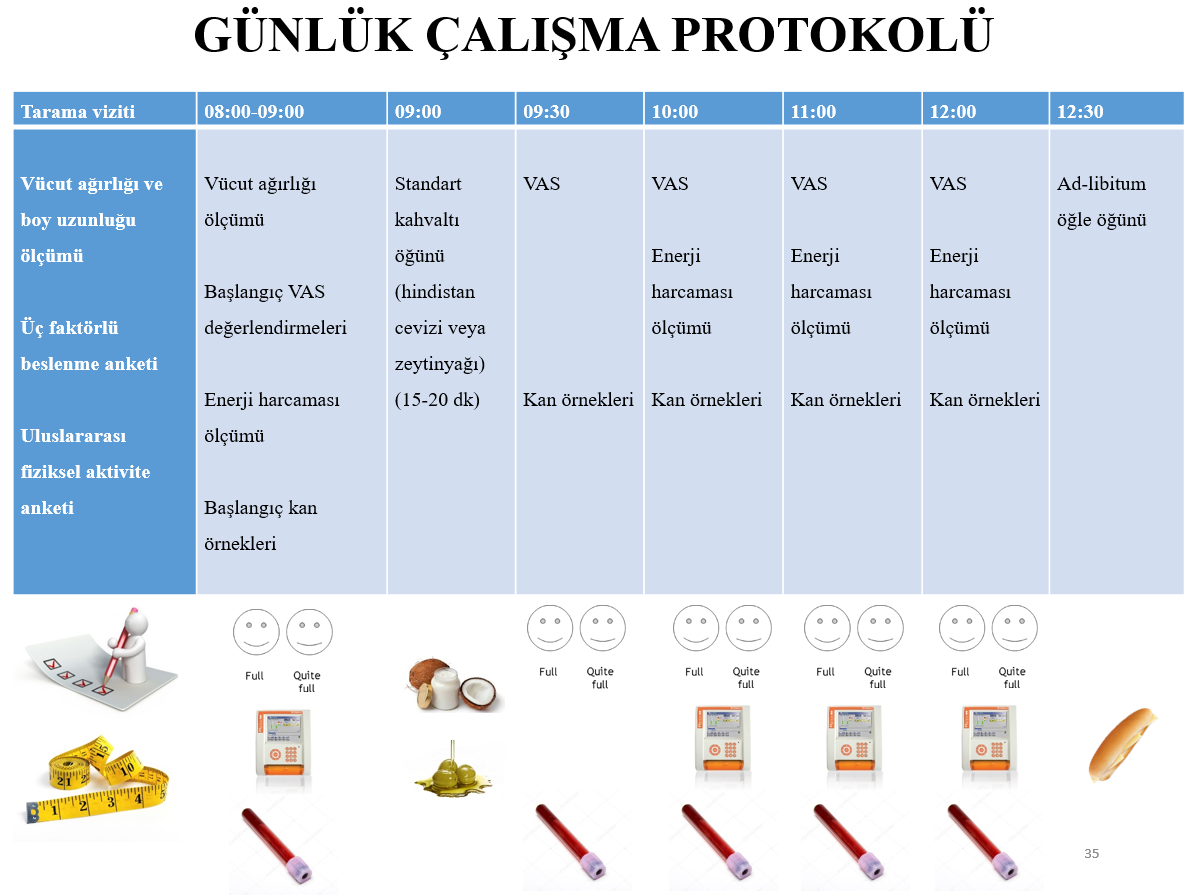

Supplement: S2 Protocol — (DOCX) [file pone.0274663.s008.docx]
